# Correlation dimension and entropy in the assessment of sex differences based on human gait data

**DOI:** 10.3389/fnhum.2023.1233859

**Published:** 2024-01-03

**Authors:** Adam Świtoński, Henryk Josiński, Andrzej Polański, Konrad Wojciechowski

**Affiliations:** ^1^Department of Computer Graphics, Vision and Digital Systems, Silesian University of Technology, Gliwice, Poland; ^2^The Research and Development Centre of the Polish-Japanese Academy of Information Technology, Bytom, Poland

**Keywords:** approximate entropy, sample entropy, correlation dimension, sex differences, gait analysis, motion capture

## Abstract

**Introduction:**

It is proved that there are differences between gait performed by females and males, which appear in movements of selected body parts. Despite numerous state-of-the-art studies related to the discriminative analysis of motion capture data, the question of whether measures of signal complexity and uncertainty can extract valuable features for the problem of sex distinction still remains open. It is the subject of the paper.

**Methods:**

Correlation dimension, as well as approximate and sample entropies, are selected to describe motion data. In the numerical experiments, the collected dataset with 884 samples of 25 females and 30 males was used. The measurements took place in the Human Motion Laboratory (HML), equipped with a highly precise motion capture system. Two variants of data representation were investigated-time series that contain joint rotations of taken skeleton model as well as positions of the markers attached to the human body. Finally, a comparative analysis between the populations of females and males using descriptive statistics, non-parametric estimation, and statistical hypotheses verification was carried out.

**Results:**

There are statistically significant sex differences extracted by the taken measures. In general, the movements of lower limbs result in greater values of correlation dimension and entropies for females, while selected upper body parts play a similar role for males. The dissimilarities are mainly observed in hip, ankle, shoulder, and head movements.

**Discussion:**

Correlation dimension and entropy measures provide robust and explainable features of motion capture data with a valuable description of the human locomotion system. Thus, beyond the importance of discovered differences between females and males, their interpretation and understanding are also known.

## 1 Introduction

There are many sex-specific differences, primarily anatomical (e.g., manifested by pelvic movements or body mass distribution), but also relating to customs (e.g., walking with high heels), which could be reflected in the way of walking. So, it is believed that sex and gender have an influence on how humans walk and on gait patterns. There are lots of studies related to the discriminative analysis of gait performed by females and males, that utilize kinematic data. Bruening et al. (2015) apply a quite straightforward approach. Ranges of motion for successive joints are calculated across gait cycles and then the mean values and standard deviations are compared. Sex differences are noticed separately for skeletal joints. Quite similarly (Fukano et al., 2018) focus on sex-based differences in the range of motion observed in talocrural flexion and subtalar rotation during walking. Barrett et al. (2008) use variability of 3D rotations for the hip, knee, and ankle, represented by the coefficient of multiple determination. Bayhan and Aydin (2018) use additionally spatiotemporal parameters—cadence, stride time, double support, stride length, step length, and walking speed—in the comparison, and Bruening et al. (2020) also take the displacements of the center of mass into consideration. The natural rhythm of locomotion is analyzed by Błaszczyk (2014), it is higher for women by about 6–9 strides/min.

In the study of Kowalski et al. (2021), the interaction of sex with state and trait physical and mental fatigue is investigated. It is assessed by the asymmetry of lateral step variability and coefficient of variation of gait speed, stride length and double limb support. Obrębska et al. (2020) focus on loadings of the knee joint while Gabriel et al. (2008)—on the stiffness of the ankle measured in the sagittal plane.

There are also approaches that utilize generic feature extraction. The most often used one is the linear dimensionality reduction technique, which maximizes the variance of output components—Principal Component Analysis (PCA). Kobayashi et al. (2016) compute PCA for the normalized motion sequences containing data of pelvic and right-lower-limb-joint angles around three axes. Discriminative abilities for the first six principal components are finally assessed. Troje (2002) takes the first four eigenpostures of PCA transformation to extract feature vector in the gender classification problem, Kastaniotis et al. (2013) compute PCA for aggregated histograms of joint rotations, Arai and Asmara (2014) use wavelet transform, while Horst et al. (2020) utilize multi-layer Convolution Neural Network instead of PCA.

There are plenty of studies related to gait-based gender differences appearing in certain disorders. Phinyomark et al. (2016) compare walking of females and males with knee osteoarthritis by means of eight discrete variables, for instance, the angle at touchdown, maximum and minimum peak angles during stance phase, and the angle at toe-off as well as using PCA. Hughes-Oliver et al. (2018) determine average values and stride-to-stride standard deviations of stride, swing, stance, and double support time intervals for ankle osteoarthritis investigation. For humans with lower back pain, movements of the thorax and lumbar spine are captured and peak amplitudes of displacements and minimal detectable changes are extracted by Bagheri et al. (2018). For multiple sclerosis patients, spatio-temporal parameters of gait, and kinematics in the sagittal plane at hip, knee and ankle joints were used by Pau et al. (2017). The stride length, gait speed, cadence, stance phase, swing phase, and double support as well as dynamic ranges of movements for the considered joints are determined. In another approach, knee and hip joints centers as well as knee joint moments calculated using inverse dynamics were used by Paterson et al. (2018). Gender and sex differences during motion activities are also studied by Di Nardo et al. (2015) and Chatain et al. (2021) using EMG and ground force reaction data, respectively.

Despite extensive research conducted on sex and gender differences of gait data, the question about the ways to discriminate walking performed by females and males remains still open. It is purposeful to explore this area as well as to propose and examine new approaches.

Remarkable possibilities to extract valuable descriptors of time sequences are given by correlation dimension and entropy. They are measures of the complexity of dynamical systems and the randomness of the observations. The correlation dimension was applied for instance in the following studies: analysis of heart rate variability in patients with dilated cardiomyopathy (Carvajal et al., 2005), assessment of EEG signals of patients with epileptic seizures (Silva et al., 1999), predicting bispectral index based on EEG (Ahmadi et al., 2010), measuring dynamics of dyad synchrony for mocap data (Buccoli et al., 2017) and decoding hand movements carried out for EMG signals (Namazi and Jafari, 2019). There are also numerous applications of approximate and sample entropies in the description of biological time-series data. Among others, they were used in the analysis of gait represented by ground reaction forces (Wu et al., 2017), in the classification of eye movement data (Harężlak and Kasprowski, 2020), the assessment of the influence of walking speed and ground slope on the gait control based on mocap sequences (Szczęsna, 2019), characterization of downhill skiing registered by inertial measurement units (Szczęsna and Janiak, 2019), examination of regularity of the center of pressure during the performance of four yoga poses (Błażkiewicz, 2020), diagnosis of unilateral knee pain (Bacon et al., 2022) as well as investigation of angular displacement when balancing on a stability platform (Becker and Hung, 2020).

It was experimentally proved, that measures of the complexity of the system controlling locomotion and randomness of movement performed by humans may give a valuable description of motion data in many challenges. Despite this, there are no studies strictly devoted to the analysis of sex differences in gait using such measures. It is a subject of the paper—the correlation dimension, as well as the approximate and sample entropies, are computed for motion capture data representing gait performed by females and males. Finally, the statistical analysis was carried out to compare the selected parameters of global distributions.

## 2 Methods

It is assumed that the system controlling human locomotion behaves like a non-linear dynamical system. Its dimensionality has an influence on the complexity of movements observed and registered by the motion capture system. To assess this dimensionality, a type of fractal dimension measure—correlation dimension—is chosen.

Thus, primarily it is required to reconstruct the phase space of the dynamical system on the basis of observations—the motion data. According to Takens' embedding theorem (Takens, 1981) time-delayed measurements are taken. For every time instant *i* vectors $x_{i}^{m}=\left[x_{i},x_{i+\tau },x_{i+2\tau },...,x_{i+\left(m-1\right)\tau }\right]$ are formed. Time delay τ is determined on the basis of the first minimum of mutual information function and embedding dimension *m* is calculated using “False Nearest Neighbors” approach. The process is described in detail by Piórek et al. (2017).

The most broadly used algorithm estimating correlation dimension was proposed by Grassberger and Procaccia (1983). It is based on the correlation sum *CS*(*r*) estimating correlation integral and defined as the fraction of pairs of points $\left(x_{i}^{m},x_{j}^{m}\right)$ in the phase space whose distances are less than *r*:


(1) $$\begin{array}{l} CS\left(r\right)=\frac{2}{\left(N-m+1\right)\left(N-m\right)}\underset{i<j}{\sum }H\left(r-∥x_{i}^{m}-x_{j}^{m}∥\right) \end{array}$$

where *N* denotes the length of the time series, *N*−*m*+1 is the number of points in the phase space, and H is the Heaviside function.

The *CS* is monotonically decreasing and at the beginning of the logarithmic scale it can be modeled by the linear function *CS*(*r*)≈*r*^*D*^. Formally, the correlation dimension (*CD*) is defined as follows:


(2) $$\begin{array}{l} CD=\underset{r\to 0}{\lim}\frac{\ln CS\left(r\right)}{\ln r} \end{array}$$

Thus, in a logarithmic scale, the linear approximation of correlation sum is determined and its slope coefficient *D* is an estimate of the correlation dimension.

There are plenty of entropy measures proposed. Two classical and chosen ones are the approximate entropy (AppEnt) and the sample entropy (SampEnt). The approximate entropy (Pincus and Goldberger, 1994) assesses the regularity and uncertainty of time series data in the following way. Using the first point of a time series of length *N* as the starting point, we form a vector ${\text{x}}_{1}^{m}$ which consists of *m* consecutive points of the time series. Repeating this procedure for the next consecutive *N*−*m* points of the time series as starting points, we form totally *N*−*m*+1 vectors ${\text{x}}_{i}^{m}$ of length *m* where 1 ≤ *i* ≤ *N*−*m*+1. The following formula determines the number of vectors similar to a given vector ${\text{x}}_{i}^{m}$ as a pattern, where the distance $d\left[{\text{x}}_{i}^{m},{\text{x}}_{j}^{m}\right]$ between vectors ${\text{x}}_{i}^{m}$, ${\text{x}}_{j}^{m}$ is defined as the maximum difference of their corresponding elements:


(3) $$C_{i}(r,m)=\frac{\sum_{j}ℋ(r-d[x_{i}^{m},x_{j}^{m}])}{N-m+1}$$

Next, the aggregation over *i* is carried out using logarithm function:


(4) $$\begin{array}{l} \Phi \left(r,m\right)=\frac{\overset{N-m+1}{\underset{i=1}{\sum }}\ln C_{i}\left(r,m\right)}{N-m+1} \end{array}$$

Finally, AppEnt is estimated as the difference of the aggregations for two consecutive values of vector length (*m* and *m*+1):


(5) $$\begin{array}{l} AppEnt\left(r,m\right)=\Phi \left(r,m\right)-\Phi \left(r,m+1\right) \end{array}$$

According to the recommendations from the literature (Richman and Moorman, 2000), in the experiments the radius of similarity *r* was calculated as 20% of the standard deviation of the entire time series and *m* was assumed to be 2.

The sample entropy (Richman and Moorman, 2000) is an unbiased statistic without an influence of self similarities. It extracts analogous features as the approximate entropy and is based on the ratio between total numbers of similar patterns (the interpretation of similarity is the same as for AppEnt):


(6) $$\begin{array}{l} SampEnt\left(r,m\right)=-\ln\frac{C\left(r,m+1\right)}{C\left(r,m\right)} \end{array}$$

where


(7) $$C(r,m)=\sum_{i<j}ℋ(r-d[x_{i}^{m},x_{j}^{m}])$$

There are extensions of chosen entropy measures introduced recently. The multiscale sample entropy (Costa et al., 2005) addresses multiscale features, the multivariate sample entropy (Looney et al., 2018) allows to assess multivariate time series, the permutation entropy (Zanin et al., 2012) directly accounts for the temporal information contained and control entropy (Bollt et al., 2009) is designed to work with non-stationary signals. Despite the fact that some of them are applicable to the faced problem and may give some advantages, at the current stage we decided to use the classical variants—approximate and sample entropies. They were extensively applied and proved to extract valuable features in numerous challenges of biomedical signal analysis.

## 3 Dataset

To compare gait performed by females and males, highly precise motion capture data were used. The acquisition took place in the Human Motion Laboratory (HML) of the Polish-Japanese Academy of Information Technology (PJAIT) (http://bytom.pja.edu.pl) equipped with the Vicon software and hardware. The collected dataset consists of 884 gait sequences of 25 females self-identifying as a woman and 30 males self-identifying as a man. The age distributions for both random samples are quite similar, as depicted in Table 1. The male part is exactly the same as the dataset used by Świtoński et al. (2019, 2021). The recording process of female data was compatible.

**Table 1:** Age [years] distribution of females and males participating in the acquisition.

**Parameter**	**Females**	**Males**
Min	22	20
Max	29	45
Average	24.9	23.1
Median	25	21
Standard deviation	2.3	5.9
Quarter deviation	3.7	2

**Figure 1:**
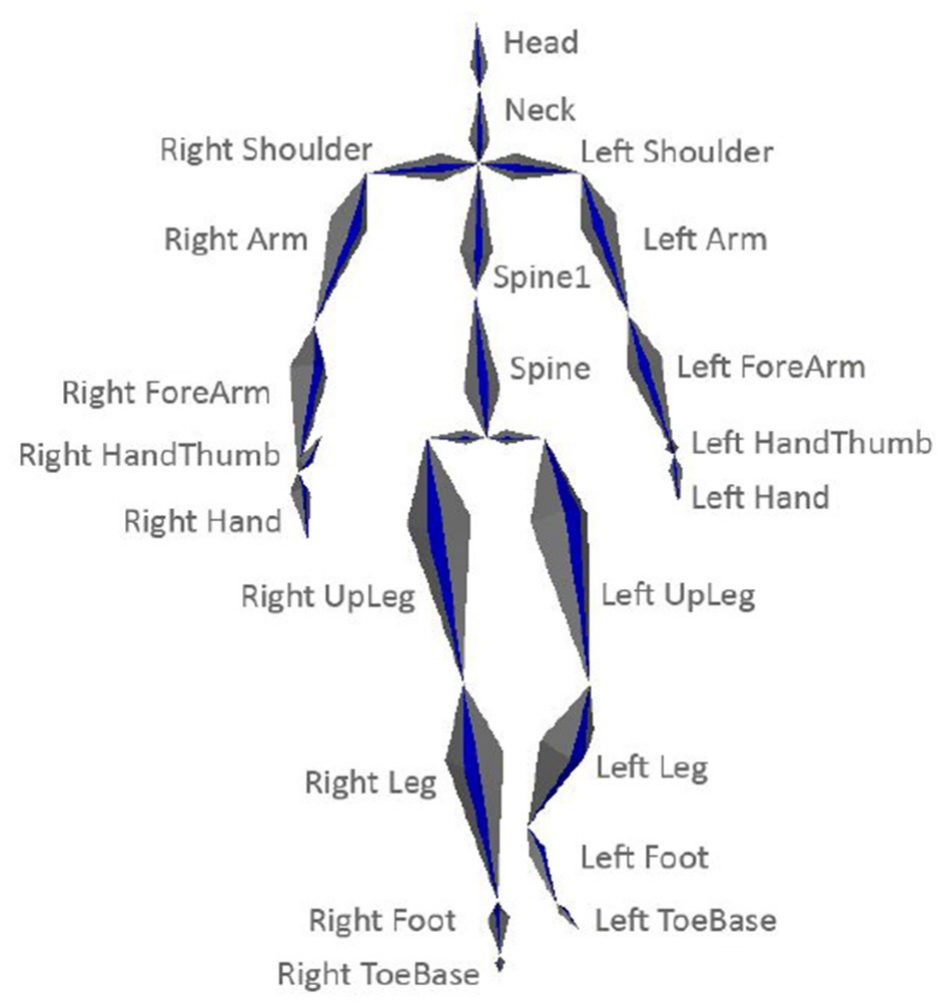
Applied skeleton model.

The default Vicon Blade skeleton, which contains 22 bone segments shown in Figure 1 was applied in the acquisition.

**Figure 2:**
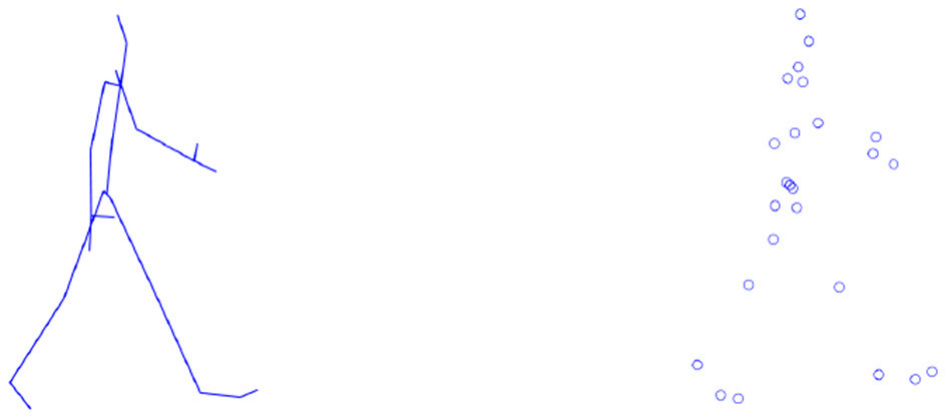
Two pose representations: rotational and point cloud.

It means that a pose is described by 3D rotational data of bone segments as well as global translation and orientation. Moreover, it can be transformed into a point cloud representation in which every joint is specified by its 3D coordinates in the global system, as visualized in Figure 2.

**Figure 3:**
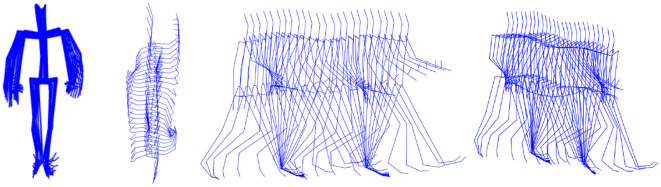
Example gait instance—front, top, side and perspective views.

The gait route was approximately five-meter-long–due to various step lengths slightly different for every participant—straight line as shown in Figure 3. Participants were asked to perform two types of gait—with natural and increased speed—that were interpreted individually. The default 100 Hz frequency was selected for the acquisition.

Due to requirements of the Vicon system, every registration starts and ends with a T-pose visualized in Figure 5B. Therefore, in further analysis, only a single gait cycle containing two adjacent steps performed by the left and right lower limbs was taken. To detect the cycles, tracking of the extremes of distances between ankles was carried out as described in detail by Świtoński et al. (2019). The middle cycle, as the most representative one, is selected. The average length of such a preprocessed time series is 108 data points.

**Figure 4:**
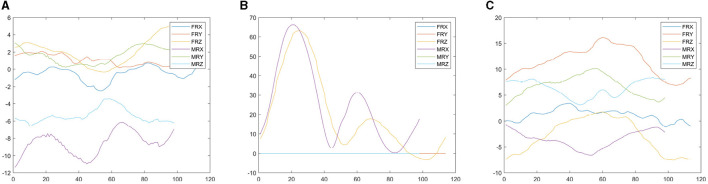
Raw mocap time series representing Euler angles triplets—RX, RY, and RZ—of Head, LeftLeg and LeftShoulder bone segments for randomly chosen female (F) and male (M) gait cycles. **(A)** Head. **(B)** LeftLeg. **(C)** LeftShoulder.

The visualization of raw mocap time series representing randomly chosen female and male gait cycles is depicted in Figure 4. It contains Euler angles triplets coding 3D rotations of bone segments.

## 4 Experimental setup

**Figure 5:**
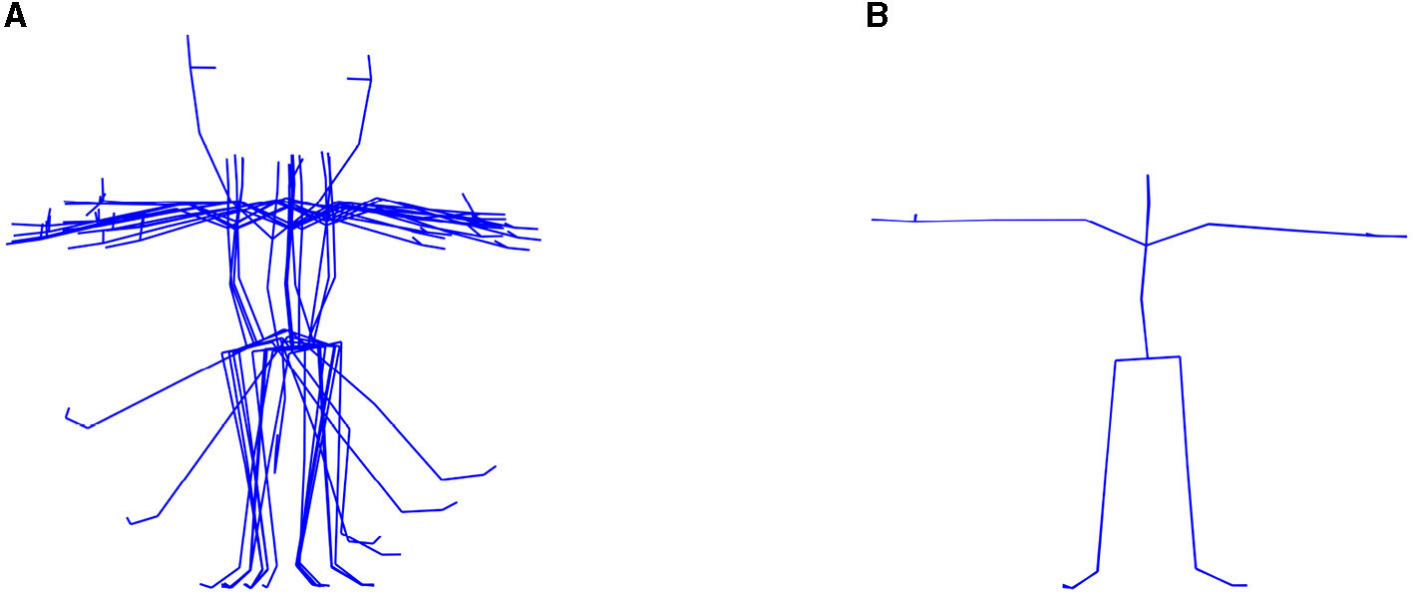
HML registration procedure. **(A)** Range of movements. **(B)** T-pose.

Multivariate mocap sequences are divided into separate time series that represent pose parameters—rotational angles or 3D markers positions. For each of them, the aforementioned measures of complexity and randomness of the signal were calculated. Thus, gait samples are described by correlation dimension as well as approximate and sample entropies determined separately for every pose parameter. Different variants of pose models were considered. In the basic one, rotations of the skeleton joints are taken. By default, they are described by Euler angles triplets with data of basic rotation around axes of the local coordinate system. However, the calibration process of motion capture acquisition, in which the local systems are determined, depends on the range of performed movements, as depicted in Figure 5A, and strict markers position on a human body. Thus, it is not fully unambiguous and results in slightly different orientations of local systems and not fully compatible models. Therefore, to simplify the description and obtain a more reliable analysis, rotations were transformed to axis-angle representation, similar to unit quaternions, and only the angle value was used.

In the second variant, point cloud representation was applied. It means that correlation dimension and entropies were determined for the sequences of the 3D joint locations in the global coordinate system.

Finally, a statistical comparative analysis for the populations of females and males, represented by random samples formed by the obtained values of taken measures, was carried out. It was aimed at exploring the differences between populations. Primarily, descriptive statistics were calculated. The mean and median values as well as standard and quarter deviations were selected. Moreover, statistical hypotheses were stated and verified to compare the parameters of the general population of females and males. The *p*-values—the probabilities of obtaining the calculated measures assuming the null hypothesis is correct—are presented.

In the second variant of the pose description, the aforementioned statistics were calculated for measures obtained for X, Y, and Z coordinates.

## 5 Results

The analysis was reduced to the parameters which describe the movements of the upper and lower limbs as well as head. The hips (LeftUpLeg, RightUpLeg), knees (LeftLeg, RightLeg), ankles (LeftFoot, RightFoot), elbows (LeftArm, RightArm), forearms (LeftForeArm, RightForeArm), shoulders (LeftShoulder, RightShoulder), and cervical (Head) joints were selected.

The obtained correlation dimension statistics for rotational data are presented in Table 2. The most remarkable difference between the populations of females and males can be noticed for time sequences representing hip movements. Significantly greater values are obtained by females. It means that the system controlling females' hips is more complex which results in more sophisticated movements. Males perform them in a simpler way. The observation is valid for both mean and median values estimated for the populations.

**Table 2:** Correlation dimension for selected joints described by an angle of rotation of skeletal data.

**Joint/Segment**	**Mean** ± **STD**		**Median** ± **QD**	
	**Females**	**Males**	**Females**	**Males**
LeftUpLeg	2.29 ± 0.64	1.97 ± 0.41	2.17 ± 0.64	1.90 ± 0.46
RightUpLeg	2.39 ± 0.69	1.96 ± 0.37	2.24 ± 0.69	1.91 ± 0.43
LeftLeg	2.04 ± 0.54	2.06 ± 0.59	1.90 ± 0.54	1.91 ± 0.66
RightLeg	2.00 ± 0.54	2.02 ± 0.53	1.89 ± 0.54	1.93 ± 0.59
LeftFoot	2.48 ± 0.59	2.35 ± 0.50	2.40 ± 0.59	2.28 ± 0.60
RightFoot	2.51 ± 0.72	2.36 ± 0.52	2.40 ± 0.72	2.28 ± 0.74
LeftArm	2.49 ± 0.68	2.44 ± 0.60	2.35 ± 0.68	2.33 ± 0.71
RightArm	2.51 ± 0.64	2.41 ± 0.61	2.39 ± 0.64	2.26 ± 0.76
LeftForeArm	2.09 ± 0.50	2.15 ± 0.52	2.02 ± 0.50	2.04 ± 0.57
RightForeArm	2.22 ± 0.51	2.26 ± 0.55	2.15 ± 0.51	2.18 ± 0.61
LeftShoulder	2.44 ± 0.66	2.61 ± 0.55	2.32 ± 0.66	2.54 ± 0.68
RightShoulder	2.48 ± 0.63	2.59 ± 0.58	2.35 ± 0.63	2.50 ± 0.67
Head	2.66 ± 0.80	2.61 ± 0.62	2.49 ± 0.80	2.55 ± 0.84

Mean values ± standard deviations as well as median values ± quarter deviations are calculated for populations of females and males.

There is a similar observation if ankles are analyzed. Once again by average, the correlation dimension is higher for the female population, but the difference in comparison to males is much smaller than for hips. The opposite dependency can be noticed for shoulder segments—this time males have greater values of correlation dimension by average. For the remaining segments, differences are low or they vary for the left and right body sides.

The mean values, as well as standard deviations of correlation dimension for time sequences with XYZ positions of bone segments, are presented in Table 3. The analysis is more troublesome and results are less clear than for rotational data. It is because the motion is decomposed into three sequences that are also related to gait direction. In general, the maximum value for all three series: X, Y, Z is most significant and should be analyzed. Moreover, the segment's position in the global system does not depend only on the rotation of the given joint. It is also an aggregation of states of preceding joints in the kinematic chain—for instance, foot position depends on the ankle as well as the hips and knee rotations. Despite this, partially quite similar observations as for rotational data can be found. Females have greater values of correlation dimension for hips and males for shoulders, but feet perform quite similarly.

**Table 3:** Correlation dimension for selected joints described by the XYZ coordinates in the global system.

**Joint/Segment**	**X**		**Y**		**Z**	
	**Females**	**Males**	**Females**	**Males**	**Females**	**Males**
LeftUpLeg	2.08 ± 0.53	1.96 ± 0.48	1.76 ± 0.33	1.81 ± 0.33	1.44 ± 0.14	1.49 ± 0.18
RightUpLeg	2.23 ± 0.71	2.02 ± 0.54	1.74 ± 0.32	1.82 ± 0.33	1.45 ± 0.14	1.48 ± 0.17
LeftLeg	1.68 ± 0.34	1.85 ± 0.37	1.43 ± 0.22	1.55 ± 0.24	0.81 ± 0.14	0.88 ± 0.14
RightLeg	1.76 ± 0.41	1.93 ± 0.39	1.38 ± 0.24	1.52 ± 0.22	0.85 ± 0.15	0.91 ± 0.13
LeftFoot	1.43 ± 0.27	1.44 ± 0.22	1.31 ± 0.22	1.33 ± 0.18	0.67 ± 0.14	0.59 ± 0.13
RightFoot	1.51 ± 0.28	1.59 ± 0.27	1.44 ± 0.28	1.43 ± 0.26	0.77 ± 0.15	0.71 ± 0.14
LeftArm	2.01 ± 0.47	2.00 ± 0.45	2.40 ± 1.04	2.53 ± 1.12	1.60 ± 0.22	1.49 ± 0.27
RightArm	2.02 ± 0.43	2.02 ± 0.40	2.40 ± 1.06	2.40 ± 1.18	1.55 ± 0.20	1.48 ± 0.23
LeftForeArm	1.98 ± 0.42	2.03 ± 0.48	2.07 ± 0.69	2.07 ± 0.65	1.41 ± 0.22	1.44 ± 0.23
RightForeArm	2.06 ± 0.48	2.02 ± 0.44	2.19 ± 0.65	2.20 ± 0.78	1.46 ± 0.22	1.50 ± 0.30
LeftShoulder	1.93 ± 0.47	1.88 ± 0.38	2.29 ± 1.16	2.51 ± 1.25	1.53 ± 0.47	1.54 ± 0.53
RightShoulder	1.93 ± 0.41	1.88 ± 0.38	2.38 ± 1.13	2.48 ± 1.28	1.61 ± 0.46	1.51 ± 0.40
Head	2.03 ± 0.47	2.08 ± 0.46	2.48 ± 1.12	2.33 ± 1.08	1.43 ± 0.40	1.45 ± 0.46

Mean values ± standard deviations are calculated for populations of females and males.

Bearing in mind that the analysis carried out for rotational data seems to be more reliable, the approximate and sample entropies were calculated only for this variant of motion data representation. The obtained statistics are depicted in Table 4. The results are roughly compatible with the ones for the correlation dimension (see Table 2) but they are even more general. The movements of lower body parts are more uncertain for females than for males—they are specified by higher entropy values. Really significant differences are obtained for hip and ankle joints, but slight ones can be noticed also for the knees. As regards the movements of the upper body parts, they are quite similar for both populations with only one exception—head movements are described by slightly higher values of entropy for males.

**Table 4:** Approximate and sample entropies for selected joints described by an angle of rotation of skeletal data.

**Joint/Segment**	**Mean** ± **STD**			
	**Approximate entropy**		**Sample entropy**	
	**Females**	**Males**	**Females**	**Males**
LeftUpLeg	0.30 ± 0.07	0.20 ± 0.05	0.27 ± 0.09	0.15 ± 0.06
RightUpLeg	0.30 ± 0.08	0.20 ± 0.05	0.27 ± 0.09	0.14 ± 0.06
LeftLeg	0.30 ± 0.07	0.27 ± 0.06	0.25 ± 0.08	0.23 ± 0.08
RightLeg	0.30 ± 0.06	0.26 ± 0.05	0.24 ± 0.07	0.22 ± 0.07
LeftFoot	0.30 ± 0.06	0.23 ± 0.05	0.28 ± 0.07	0.20 ± 0.05
RightFoot	0.31 ± 0.06	0.23 ± 0.04	0.29 ± 0.07	0.20 ± 0.05
LeftArm	0.34 ± 0.08	0.29 ± 0.09	0.31 ± 0.11	0.25 ± 0.11
RightArm	0.34 ± 0.08	0.30 ± 0.08	0.32 ± 0.11	0.26 ± 0.10
LeftForeArm	0.26 ± 0.05	0.24 ± 0.07	0.20 ± 0.07	0.19 ± 0.09
RightForeArm	0.28 ± 0.05	0.26 ± 0.08	0.20 ± 0.06	0.21 ± 0.10
LeftShoulder	0.33 ± 0.06	0.33 ± 0.08	0.31 ± 0.09	0.30 ± 0.11
RightShoulder	0.34 ± 0.06	0.33 ± 0.09	0.31 ± 0.09	0.31 ± 0.12
Head	0.42 ± 0.11	0.46 ± 0.13	0.45 ± 0.17	0.49 ± 0.21

Mean values ± standard deviations are calculated for populations of females and males.

Finally, the statistical hypotheses were stated and verified. They address discriminative features of knees, ankles, shoulders, and arms movements in respect to correlation dimension and both measures of entropy calculated for rotational data.

**Figure 6:**
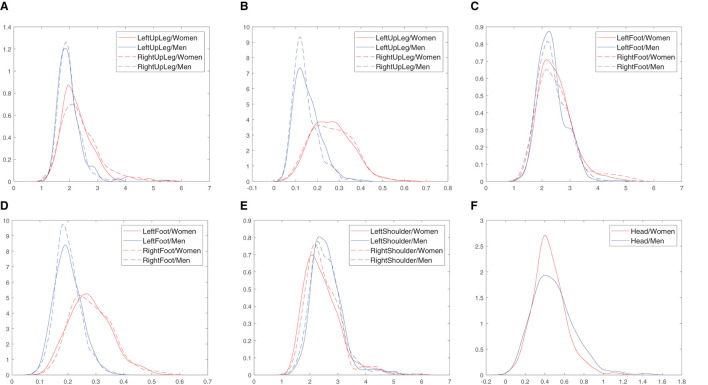
Kernel density estimation of correlation dimension (CD) and sample entropy (SampEnt) of the hip, ankle, shoulder and head movements calculated separately for the populations of females and males. **(A)** CD, Hips. **(B)** SampEnt, Hips. **(C)** CD, Ankles. **(D)** SampEnt, Ankles. **(E)** CD, Shoulders. **(F)** SampEnt, Head.

In the first stage, the normality of distributions was examined. The Shapiro-Wilk test (Stąpor, 2020) was selected. The obtained *p*-values presented in Table 5 for the most cases are very low (< 0.001)—the null hypotheses as being very unlikely are rejected. It means that the normal distribution cannot be assumed. Therefore, non-parametric estimation using kernel based method (Stąpor, 2020) was carried out to visualize the distributions and differences between them. The example outcomes are visualized in Figure 6. The same observations can be made—there are shifts between the distributions for females and males. For the LeftUpLeg and RightUpLeg (hips), greater values are more probable for females in respect to both measures (CD, Figure 6A, and SampEnt, Figure 6B). There is a similar but more significant difference for the LeftFoot and RightFoot (ankles), but only if sample entropy is considered (Figures 6C vs D). As regards the males, shoulder movements result in greater correlation dimension values (Figure 6E) and head movements are associated with greater values of sample entropy (Figure 6F).

**Table 5:** The *p*-values of Shapiro-Wilk test examining normality of distribution of correlation dimension (CD), approximate and sample entropies (AppEnt, SampEnt) calculated for the movements of selected joints in populations of females and males.

**Joint/Segment**	**CD**		**AppEnt**		**SampEnt**	
	**Females**	**Males**	**Females**	**Males**	**Females**	**Males**
LeftUpLeg	< 0.001	< 0.001	< 0.001	< 0.001	< 0.001	< 0.001
RightUpLeg	< 0.001	< 0.001	< 0.001	< 0.001	< 0.001	< 0.001
LeftFoot	< 0.001	< 0.001	0.203	0.181	< 0.001	< 0.001
RightFoot	< 0.001	< 0.001	0.001	0.010	< 0.001	< 0.001
LeftShoulder	< 0.001	< 0.001	0.318	< 0.001	< 0.001	< 0.001
RightShoulder	< 0.001	< 0.001	0.024	< 0.001	< 0.001	< 0.001
Head	< 0.001	< 0.001	< 0.001	0.140	< 0.001	< 0.001

To finally assess the observed discrimination features, the Mann-Whitney-Wilcoxon test (Gürsoy et al., 2015) was applied. The null hypothesis assumes that the cumulative distribution functions for compared global distributions *F*(*x*) (females) and *M*(*x*) (males) are the same against the following alternative hypotheses:

a) right tailed: *F*(*x*)≥*M*(*x*),
b) left tailed: *F*(*x*) ≤ *M*(*x*).

The results obtained are depicted in Table 6. In most cases, the null hypotheses are rejected as being very unlikely (*p*-value < 0.001). It confirms statistical significance that movements of lower limbs in hips and ankles are described by greater values of correlation dimension and entropies for the population of females, as previously noticed. In the case of males, the shoulder movements are associated with greater values of correlation dimension, while head movements—with greater values of both entropy measures.

**Table 6:** The *p*-values of the Mann-Whitney-Wilcoxon test for populations of females and males described by correlation dimension (CD) as well as approximate and sample entropies (AppEnt, SampEnt) calculated for the movements of selected joints.

**Joint/Segment**	**Tail**	**CD**	**AppEnt**	**SampEnt**
LeftUpLeg	Right	< 0.001	< 0.001	< 0.001
RightUpLeg	Right	< 0.001	< 0.001	< 0.001
LeftFoot	Right	< 0.001	< 0.001	< 0.001
RightFoot	Right	0.007	< 0.001	< 0.001
LeftShoulder	Left	< 0.001	0.964	0.998
RightShoulder	Left	< 0.001	0.999	0.891
Head	Left	0.311	< 0.001	0.006

In total, the maxima of the obtained values for the correlation dimension, approximate, and sample entropies are 6.3, 0.81, and 1.6, respectively.

## 6 Conclusions

The existence of differences in the gait of females and males has been repeatedly emphasized in the literature for a long time (Murray et al., 1966, 1970; Mather and Murdoch, 1994; Troje, 2002), with pelvis and torso being key parts of the human body when it comes to pointing out these differences. They mainly concern the frontal plane–Bruening et al. (2015) indicate a greater pelvic obliquity range of motion in females than in males and the opposite situation when it comes to torso sway. Movement of the pelvis in the transverse plane also tends to be greater in females than in males (Bruening et al., 2015). A similar observation concerns the range of ankle motion in the sagittal plane (Bruening et al., 2020).

Our results obtained using methods referring to the complexity and regularity of the analyzed signal seem to be consistent with these observations. The movements of lower limbs—particularly hips and ankles—are more complex and uncertain for the population of females. It results in greater values of correlation dimension as well as entropies. In the case of upper body parts for some of them, mainly shoulders, males have higher values of the taken measures, but mostly they are quite similar. It is consistent with the general perception of how females and males walk and the attention paid by a typical observer.

It is possible to aggregate 3D rotations to a single value that preserves features related to complexity and randomness. The discriminative analysis conducted on the basis of time sequences containing the XYZ coordinates of selected body parts is more troublesome.

The values of the correlation dimension and both entropies do not discriminate human body movements in exactly the same way, but they are compatible and correlated. They mostly confirm the same observations and provide robust and interpretable features of motion capture data.

Both entropies extract almost identical values. Thus, the known drawback of the approximate entropy that it is biased and lacking relative consistency, does not have crucial meaning in case of the analysis of motion capture data.

The collected dataset contains gait sequences performed only by females self-identifying as woman and males self-identifying as man. Thus, the influences of sex and gender on differences noticed can not be distinguished.

The main limitation of the research is related to the time series lengths. For both correlation dimension and entropies, the estimation's reliability depends on the sequence's duration. It is feasible to process whole registered sequences, but it means starting T-pose, required by the Vicon system, is also the subject of the analysis. Another possible variant is taking more than one gait cycle—in most cases, every recording contains three. However, the first and last ones correspond to the start-up and end-up phases. The most natural gait cycle is the one extracted from the middle of the sequence, which is taken in our computations. Moreover, there are no strict limits on the time series duration for the estimation of correlation dimension and entropy. Pincus (1995) recommends that AppEnt could be used even in case of 75 to 100 data points. It is also confirmed by Delgado-Bonal and Marshak (2019). This length condition is met by our time series representing a single gait cycle, recorded at a frequency of 100 Hz.

## Data availability statement

The original contributions presented in the study are included in the article. Further inquiries on data can be directed to the corresponding author.

## Ethics statement

Human gait data used were collected by a non-invasive Vicon system by the certified staff according to regulations provided by the Vicon company and approved by PJAIT academy. There were no methods performed on humans in any invasive way. Further ethical review and approval was not required for the study on human participants in accordance with the local legislation and institutional requirements. The participants provided their written informed consent to participate in this study.

## Author contributions

Methodology proposal and manuscript preparation: AŚ and HJ. Formal analysis and results analysis: AŚ, HJ, AP, and KW. Experiments implementation: AŚ. All authors contributed to the article and approved the submitted version.
